# Recent developments in genetic/genomic medicine

**DOI:** 10.1042/CS20180436

**Published:** 2019-03-05

**Authors:** Rachel H. Horton, Anneke M. Lucassen

**Affiliations:** Clinical Ethics and Law, Faculty of Medicine, University of Southampton, Southampton, United Kingdom

**Keywords:** genetics, genomics, ethics

## Abstract

Advances in genetic technology are having a major impact in the clinic, and mean that many perceptions of the role and scope of genetic testing are having to change. Genomic testing brings with it a greater opportunity for diagnosis, or predictions of future diagnoses, but also an increased chance of uncertain or unexpected findings, many of which may have impacts for multiple members of a person’s family. In the past, genetic testing was rarely able to provide rapid results, but the increasing speed and availability of genomic testing is changing this, meaning that genomic information is increasingly influencing decisions around patient care in the acute inpatient setting. The landscape of treatment options for genetic conditions is shifting, which has evolving implications for clinical discussions around previously untreatable disorders. Furthermore, the point of access to testing is changing with increasing provision direct to the consumer outside the formal healthcare setting. This review outlines the ways in which genetic medicine is developing in light of technological advances.

## Introduction

The past two decades have seen major shifts in our technical ability to sequence genetic information at scale. Historically, genetic testing tended to consist of either highly detailed molecular testing of nominated single genes, or broad genome-wide dosage screening at low resolution, for example karyotyping [1,2]. Genome sequencing was too slow and too expensive to be used in clinical contexts: for example the Human Genome Project, which was 99% complete in 2004, cost three billion dollars and took 13 years to sequence [3].

More recently, advances in sequencing technology have made it possible to undertake broad genetic testing on an individual patient basis within a clinically useful timeframe, via exome and genome sequencing. Exome tests sequence the entire protein-coding region of the genome, representing less than 2% of the genome but containing approximately 85% of known disease-causing variants [4]; genome sequencing encompasses the exome but also sequences all the non-protein-coding DNA. Initially implementation of such tests was via clinical research studies such as the Deciphering Developmental Disorders project [5], but more recently exome sequencing has been utilised as a clinical diagnostic test [6]. Genome sequencing is also due to transition to being available as a standard NHS test in June 2019, having previously only been available via initiatives such as the 100,000 Genomes Project [7].

Sequencing technology has improved in depth as well as breadth, and this has been of importance in better understanding cancer. The ability to sequence cancer genomes has led to rapid identification of driver mutations and has helped to work out the complex relationships between different cancer subclones over space and time, demonstrating the enormous heterogeneity of cancers and the difficulty of successfully treating them [8]. As sequencing techniques have advanced to the level where tiny amounts of tumour or individual cells can be sequenced, it has been possible to identify previously unknown mutational mechanisms, such as chromothripsis^1^ [9] and kataegis^2^ [10].

However, our ability to generate genomic data has substantially outstripped our ability to interpret its significance for an individual, and while improvements in genomic technology are in many cases driving improvements in healthcare, we are also encountering new problems as genomic testing shifts into the clinical setting. The Global Alliance for Genomics and Health (GA4GH) predicts that by 2025, over 60 million people will have had their genome sequenced in a healthcare context [11], but pathways for managing the output from genome sequencing are still in their infancy. The detailed but unfocused approach of genomic tests gives opportunities to answer questions that go beyond the problems that led to a patient having a test. However, deciding which of the multitude of possible outputs from genomic tests should be considered a ‘result’ at any given time is very challenging, not least because the links between many genetic variants and diseases are often unproven or poorly understood [12]. Multidisciplinary input and collaboration are increasingly key to interpreting the significance of genomic results. This review discusses the developments in practice that are evolving as a result of increasing use of genomic technologies.

## New disease gene discovery and changing concepts of diagnosis

Exome and genome sequencing are powerful diagnostic tools – for example the Deciphering Developmental Disorders project, which recruited patients with severe undiagnosed disorders (who had generally already had any currently available diagnostic genetic testing), achieved a 40% diagnosis rate via trio exome sequencing for the first 1133 family trios in the study [13]. The search for a diagnosis has often been described as a journey [14], with parents of children with rare genetic disorders anticipating that a diagnosis may guide treatment, prognosis, acceptance and social support [15]. However, identification of new rare disease genes may be changing the impact of receiving a diagnosis, and in many cases very little is known about the long-term effects of newly identified genetic conditions.

Historically when making a genetic diagnosis, it has usually been possible to give families some information regarding prognosis, and to provide some parameters as to what to expect for the future, based on previous experience of what has happened for other children affected by the same condition. Now, while in some situations due to strong phenotypic match it is possible to be confident that a child’s rare disease has been caused by pathogenic variants in a recently described rare disease gene, often this provides little information about a child’s future.

We are increasingly in the position of learning about the effects of possible disease-causing variation(s) in a gene through meeting the patients in whom such genetic changes have been discovered. Often these changes will be in a gene newly thought to be linked to developmental disorders and there will be little, if any, published literature to draw on. We then have to speculate whether the genetic change detected is the cause of our patient’s health problems, and whether any additional difficulties that have happened for our patient that have not yet been noted in other patients with changes in the same gene are an extension of the phenotype of the newly described disease gene, or coincidental. In situations like this, we are often unable to give people information about what a new diagnosis might mean for them or their child in the longer term.

This has led to patient support and awareness groups taking on an increasingly important role [16], as families gather to share their lived experience of newly diagnosed rare genetic conditions, in turn informing clinical services. For example, the charity Unique works with families and professionals to develop specialist information relating to many rare and newly described genetic conditions, and to gather information about their long-term effects, increasing awareness and understanding of what it is like to live with rare genetic conditions. The rapidity with which such information can be gathered is also exemplified by the work of the PURA Syndrome Foundation: in 2014 the first patients with a rare condition called PURA syndrome were described in the medical literature [17]. Shortly afterwards the PURA Syndrome Foundation was established which has catalysed links between families, clinicians and researchers, greatly improving the speed and quality of research into the condition [18].

The agnostic approach of exome and genome sequencing is also challenging our previous concepts of existing genetic diagnoses, when apparently pathogenic variants are found in well-described disease genes but the patient’s clinical picture falls outside the boundaries of what we would conventionally expect for a patient affected by that particular genetic condition. For example, loss-of-function variants in *SOX2* are known to cause anophthalmia and microphthalmia in addition to other phenotypes such as developmental delay and structural brain anomalies. Eye abnormalities were thought to be a key feature of *SOX2*-related disorders, and so *SOX2* would only be requested as a genetic test in patients who had absent or small eyes. Recently, via ‘genotype-first’ approaches, loss-of-function *SOX2* variants have been found in people with developmental delay but without anophthalmia or microphthalmia, broadening the phenotypic spectrum associated with this gene [19]. Case Study 1 shows a further example where exome testing has extended previous perceptions of the clinical scope of a genetic condition.

Case Study 1 **Redefining our understanding of genetic conditions (fictional case based on Eggens et al. [20])**

An 8-year-old girl was referred to clinical genetics in order to investigate her progressive weakness. She had been floppy as a baby and from the age of 5 years had developed worsening limb weakness with frequent unusual movements, and difficulty in swallowing. Serial brain scans had shown progressive cerebellar atrophy.

Exome testing found that she was homozygous for a variant predicted to disrupt the function of *EXOSC3*, a gene associated with pontocerebellar hypoplasia. This diagnosis had never been thought of as she did not have one of the defining characteristics: pontine hypoplasia. Her clinical picture also seemed atypical for this condition – most children with pontocerebellar hypoplasia do not survive infancy.

However, recent research has shown genotype–phenotype correlations in *EXOSC3*-mediated pontocerebellar hypoplasia – patients homozygous for p.D132A variants (like this patient had) tend to have a milder clinical course and preservation of the pons. This genetic explanation fitted well in retrospect, but would not have been considered in advance of the exome test.

**Key messages**

- Many well-recognised genetic conditions may have a wider spectrum of effects than previously thought.
- Patients with genetic conditions identified via genomic tests may not conform to the pattern we expect based on experience of patients with the same condition identified via single gene testing. It can be very difficult to be sure whether this reflects an incorrect diagnosis, or a wider disease spectrum than previously recognised.

In many cases, our understanding of why the same genetic condition may be expressed so differently among different people is at an early stage, and this often makes genetic counselling very challenging, particularly in the prenatal setting. For some genetic conditions, it is becoming possible to provide more personalised risk estimates, based on combining knowledge of a person’s genetic diagnosis, with analysis of other factors that may influence their risk. Personalisation of risk in this way has generally been crude and reliant on clinically obvious characteristics: for example, men with pathogenic *BRCA* variants have a lower risk of developing breast cancer than women with pathogenic *BRCA* variants. More recently, genetic testing is being developed to complement ‘key’ genetic test results to provide an increasingly refined personal risk. For example, use of a polygenic risk score using breast cancer and ovarian cancer susceptibility SNPs identified via population GWAS showed large differences in absolute cancer risks between women with pathogenic *BRCA* variants with higher compared with lower polygenic risk score values [21]. This has yet to translate into routine clinical practice, but has the potential to help women with pathogenic *BRCA* variants make more informed decisions about how and when to manage their cancer risk.

## The downsides of improved sensitivity: increased uncertainty in what tests mean

The prior probability of any one variant identified via genome sequencing being causative for a patient’s rare disease is extremely low. Attempts to catalogue human genetic variation, for example via the 1000 Genomes Project, show that a typical human genome differs from the reference human genome at 4.1–5 million sites [22]. Most of these variations will be entirely benign, some may subtly impact on risk of various common diseases, and a very small number will have the potential to cause serious disease either in an individual, or in their children (potentially in combination with variants inherited from their partner).

Genome sequencing identifies the majority of these variants, which then need careful filtering to produce a meaningful output. This has required a significant change in mindset from an era when most variants were identified in the context of carefully chosen single gene sequencing, and so had a much higher prior probability of being causative. There is an increasing shift towards a view that variants should be ‘innocent until proven guilty’ [23], but there is a lack of consensus regarding how to translate this principle into clinical practice.

There is also considerable discrepancy in how different genetics laboratories interpret the same variants. International guidelines for variant interpretation are helpful but insufficient to remove a great deal of noise when attempting to assign significance to particular findings [24]. This was illustrated in a recent study comparing variant classification among nine genetic laboratories: although they all used the same guidelines, only 34% of variants were given the same classification by all laboratories, and 22% of variants were classified so differently that different medical interventions would be recommended [25]. At a lower resolution level, even being sure of the relationship between genes and diseases is often difficult. For example, curation of the 21 genes routinely available on Brugada syndrome gene panels using the ClinGen gene curation scoring matrix found that only one of these genes was definitively linked to Brugada syndrome [26]. Our improving knowledge of variant interpretation leaves us with a difficult legacy, with many patients having been diagnosed incorrectly with genetic conditions. The effects of this can be far-reaching and difficult to undo, as illustrated by Case Study 2.

Case Study 2 **The legacy of incorrect diagnosis (case reported by Ackerman et al. [27])**

A teenage boy died suddenly and genetic testing was then undertaken for his brother, resulting in the finding of a rare variant in *KCNQ1*. On the basis of this test, the living brother was diagnosed with long QT syndrome, and the teenage boy’s sudden death was attributed to long QT syndrome. The living brother had an implantable cardioverter defibrillator inserted, and via cascade genetic testing over 24 relatives were diagnosed as having long QT syndrome, despite having normal QT intervals on ECG.

However, subsequent examination of post-mortem samples found that the boy who died had cardiac features inconsistent with long QT syndrome, did not have the *KCNQ1* variant found in the wider family, and instead had a clearly disease-causing *de novo* variant in *DES*, a gene linked to cardiomyopathy.

**Key messages**

- It is very important to consider whether the clinical picture fits when evaluating variant significance: genetic variants will usually only predict disease well if found in the context of a medical or family history of the relevant disease.
- Incorrect (or inappropriately deterministic) genetic test interpretation can affect the clinical care of a whole family, not just the person being tested.

Although this suggests that we need to be very cautious in making firm genetic diagnoses, it is difficult to know where the threshold should lie for communicating genetic variation of uncertain significance. There is some evidence that people find receiving a variant of uncertain significance surprising and disturbing, and some people misinterpret it as being definitely pathogenic or definitely benign [28]. However, there is also evidence that many people have a strong desire to receive a broad range of results from genetic testing, including uncertain results, and are uncomfortable with the idea that decisions about non-disclosure might be made without involving them [29].

The fear is that disclosure of uncertain variants will lead to over-diagnosis and over-management, with variants inappropriately being treated as if pathogenic. Excessive and inappropriate interventions (not to mention anxiety and distress) might then cascade through families, going against one of the fundamental principles of medicine to ‘first do no harm’. However, we also fear missing something or being accused of ‘hiding information’. The result is that we tend to end up in purgatory, documenting uncertain variants on lab reports (though sometimes not) and having lengthy conservations with patients about them (though sometimes not), then tacking on a caveat that ‘maybe this means nothing’. This nominally shifts the responsibility to the next person in the chain but feels unsatisfactory for all concerned.

## Uncertainty when to stop looking and what to communicate

Another issue arising from improved sensitivity is the ability to find genetic variants that are unrelated to the clinical problem that a patient presents with, but that may be relevant for their health in other ways. This may be viewed as positive or negative, but working out how to handle this information raises difficult questions. In 2013, the American College of Medical Genetics and Genomics (ACMG) suggested that laboratories should automatically seek and report pathogenic variants in 56 genes associated with ‘medically actionable’ conditions when performing clinical sequencing [30]. The main rationale was the potential to benefit patients and families by diagnosing disorders where preventative measures and/or treatments were available, with the aim of improving health. However, these recommendations proved controversial. The main debate at the time centred around whether patients should have a right to choose not to know such information [31]. Subsequent questions about the role of clinicians in offering additional findings, what constitutes a ‘medically actionable’ finding, and what is the predictive value of such findings in the absence of a phenotype or family history of the relevant disorder, are yet to be fully addressed.

Analysis of data from the 1000 Genomes cohort demonstrated that approximately 1% of ‘healthy’ people will have a ‘medically actionable’ finding in one of the 56 genes [32]. However, what this might mean on an individual basis is often unclear. Most of our knowledge regarding the effects of variation in any given gene has been gathered by observing people who have been identified as having variants in the gene because they were tested as they had a personal history or family history of disease, biasing the sample from which our conclusions are drawn. It is less clear what it might mean to find, for example, an apparently pathogenic variant in a gene linked to cardiomyopathy in a person with no personal or family history of heart problems. This has important implications for ‘cascade screening’, where relatives of a patient affected by a condition with a known genetic cause are offered testing to see whether they have the disease-causing genetic variant that was found in their clinically affected family member (meaning that they may also be at risk of developing the disease). To what extent should testing and subsequent screening be offered in a family based on an incidental finding of a genetic variant thought to be predictive of a particular condition, if there is no clinical evidence that anyone in the family, including the person in whom the genetic variant in question was first identified, is actually affected by it?

Broad genomic testing also has the potential to detect carrier status for recessive and X-linked conditions. From population studies, we know that being a carrier for a genetic condition is very common. For example, a gene panel testing carrier status for 108 recessive disorders in 23453 people found that 24% were carriers for at least one of the 108 disorders, and 5.2% were carriers for multiple disorders [33]. On a disorder-by-disorder status, being a carrier for a genetic condition is very rare (with notable exceptions such as haemochromatosis and cystic fibrosis), but when considered collectively, it is ‘normal’ to be a carrier for a genetic condition. For most people, being a carrier will have no impact on their life at all. However, if their partner happens to be a carrier for the same condition then the implications could be very profound, as each of their children would have a one in four chance of being affected by the genetic condition. This is particularly relevant for couples who are known to be biologically related [34], and couples with common ancestry, as they will have a higher chance of both being carriers for the same recessive condition. Carrier screening for various autosomal recessive diseases has been available in some instances for many years, for example screening for carrier status for Tay–Sachs disease for people of Ashkenazi Jewish ancestry has been offered since the 1970s [35,36]. More recently, advances in technology have led to development of expanded carrier screening tests, which check carrier status for multiple diseases simultaneously and are often less targeted towards particular genetic populations [37].

The increased scope of carrier screening, combined with the recognition that it is very common to be a carrier for one or more recessive genetic conditions, has led to an increasing move to consider carrier results for recessive genetic conditions on a couple basis, where carrier status is only communicated if it would be relevant in the context of a particular relationship (i.e. if both people in a couple are carriers for the same condition) [38]. This avoids pathologising the status of ‘being a carrier’, recognising that most of us are carriers for some genetic conditions, and conserves resources for genetics services by not flooding the system with large volumes of individual carrier results, most of which will be meaningless in the context of that individual’s life. Objections to this approach are that by not communicating individual carrier results, a person would not know this information for future relationships, and their family could not access cascade screening to see whether they are also carriers. However, these objections could be obviated by widespread adoption of couple carrier testing – a person (or their close relatives) could find out their carrier status if relevant when they next had a couple carrier test in the context of their new relationship. In some ways, this could be seen as comparable with management of infectious disease – lots of healthy people carry MRSA, but very few die of MRSA infection. People are therefore screened at times when they might be especially vulnerable to becoming unwell from MRSA, or when they might pass it on to others at risk, for example when admitted to hospital, rather than being tested at random points when they are generally well.

## The expanding remit and availability of genetic technology

### ‘Acute genetics’

For many years, clinical genetics input has at times influenced acute care, for example in diagnosing trisomies in the neonatal period, or informing the care of babies born with ambiguous genitalia. However in many circumstances, the key contribution of clinical genetics was in providing a post hoc explanation for serious medical problems, rather than in influencing treatment decisions on a real-time basis. This is changing as the availability of exome and genome sequencing increases, as shown by Case Study 3. A recent study in a neonatal intensive care unit in Texas studied outcomes for 278 infants who were referred for clinical exome sequencing, and found that 36.7% received a genetic diagnosis, and medical management was affected for 52% of infants with diagnoses [39]. There is increasing evidence that this approach is cost-effective: for example, a prospective study of exome sequencing for infants with suspected monogenic disorders found that standard care achieved an average cost per diagnosis of AU$ 27050, compared with AU$ 5047 for early singleton exome sequencing [40]. Similarly, ‘real-time’ genetic and genomic testing is making an impact in cancer treatment, where in many cases testing is available to help guide treatment choices by identifying actionable genetic variants in tumours that may respond to specific therapies [41,42].

Case Study 3 **Insights from exome testing transforming a clinical course (case from Wessex Genomic Medicine Centre [43])**

A young woman was referred for exome testing having spent months in a coma. From childhood she had experienced sensory problems, and as a young adult she had gone on to develop seizures which deteriorated into status epilepticus, necessitating ventilation on intensive care.

After 3 years during which all other avenues had been explored, analysis of her exome was proposed. An unexpected diagnosis of pyridoxine-dependent epilepsy was found; this had not previously been considered as classically it causes seizures in the first few months of life. She began treatment with pyridoxine (vitamin B_6_). From that point on she had no further seizures and her clinical situation transformed. Over a 6-month period she was weaned off all of her anti-epileptic drugs, and was able to return to a normal life.

**Key message**

- Exome or genome tests have the potential to make an enormous difference to clinical care and to people’s lives.

### Pharmacogenomics

As well as guiding treatment choice, genetic testing will increasingly influence what doses are prescribed, and whether medications are considered unsuitable in view of a high risk of an adverse reaction. Around the time that the Human Genome Project was completed, there was considerable excitement about the possibility of genetic testing guiding use of medication in the clinic [44,45]. The potential of genotype-driven drug dosing has for the most part yet to be realised, in part because the interaction of the genetic factors involved is sometimes complex, and in part because environmental factors may also have a significant impact on how a person responds to a drug. For example, genotype-driven prescription of warfarin, which has notoriously wide inter-individual variation in dosage requirements, largely remains in the realm of research [46].

However, for some drugs, pharmacogenomics has already had a significant impact in reducing morbidity and mortality. For example, when the antiretroviral drug abacavir was first introduced, approximately 5% of the people treated developed an idiosyncratic hypersensitivity reaction that could be life-threatening on repeated exposure to the drug [47,48]. Research established that immunologically confirmed hypersensitivity reactions to abacavir only occurred in people with the HLA-B*5701 allele, and a clinical trial went on to show that pre-screening patients to check that they did not have HLA-B*5701 prior to starting the drug led to no confirmed hypersensitivity reactions in the pre-screened arm, while 2.4% of the unscreened patients had reactions [49]. Patients are now screened for HLA-B*5701 as standard before starting abacavir treatment [50]. Similar screening is likely to become more widespread as we learn more about genetic risk factors for adverse drug reactions. For example, there are increasing suggestions that the mitochondrial variant m.1555A>G should be checked in patients with cystic fibrosis in order to guide antibiotic treatment choices, in view of the evidence that people with this variant may develop hearing loss when exposed to aminoglycosides [51].

### Evolving options in prenatal genetics

Genetic testing is also being used more extensively in the prenatal setting, in part because of developments in non-invasive prenatal testing and diagnosis, which allow genetic screening or testing of a developing pregnancy by doing a blood test for the mother [52]. This removes the risk of miscarriage associated with conventional prenatal tests (chorionic villus sampling or amniocentesis). While this is in some ways a stride forward, it raises various ethical issues, as the technical test safety may lead to such testing becoming viewed as routine. This raises the concern that couples will give less careful consideration as to whether they really want to know the results before having such tests, and that women may feel that there is an expectation that they should have testing. The worry is that this could potentially lead to people feeling under pressure to terminate pregnancies in response to genetic test results (including in situations where the clinical implications of the results may be far from clear) [53].

### Widening access to genetic testing within healthcare

The expanding options for genetic testing and the escalating expectation for quick results to drive clinical management mean that testing provision is increasingly being pushed out of highly specialised genetics centres into mainstream medicine. For example, many women with ovarian cancer will now be offered *BRCA* testing via their oncology team, and only referred to genetics if needed based on the test results [54]. Genetics appointments now frequently focus on interpretation of tests already done, working out if the test outcome seems to match the clinical problem, and arranging testing and surveillance for family members.

### The rise of direct-to-consumer genetic testing

As clinical services have increasingly grown to expect and demand genetic answers for patients with complex health problems, on a broader societal level the hunger for genetic information also seems to be increasing. However this is occurring in the context of a public discourse about personalised/precision medicine and genetics that tend to enthusiastically promote it in a very optimistic light, rarely dwelling on potential concerns and limitations, and therefore potentially sculpting inappropriate expectations from technology that is still being developed [55].

Direct-to-consumer tests currently sit outside much of the regulation that governs clinical genetic testing, but claim to provide insight into issues as diverse as ancestry, nutrition, athletic ability, and child talent [56]. Many testing providers also claim to help provide insight on health, though the information provided by many direct-to-consumer companies is far from comprehensive. For example, a recent analysis of 15 direct-to-consumer genetic testing companies advertising to U.K. consumers found that none of them complied with all the U.K. Human Genetics Commission principles for good practice regarding consumer information [57]. There are also examples that might make us reflect sceptically on the value of these tests – for example a case where a family sent a sample from their dog to a direct-to-consumer testing company designed to provide insights on people’s genetic ‘superpowers’ and received a report which did not mention that the sample was not human but conjectured that the client would be talented at basketball [58].

‘DIY genetics’ has also risen in popularity, with people asking for raw data from direct-to-consumer companies then processing this themselves via third-party interpretation services, as discussed in Case Study 4. Approximately 40% of genetic changes in direct-to-consumer test raw data sent for clinical confirmation are false positives [59], but this is often not appreciated by customers or the doctors they may subsequently visit, leading to anxiety and often inappropriate medical interventions [60]. However, clearly many people see a value in receiving genetic information and are prepared to pay for this. This marks a shift from genetic testing in order to explain health problems or for people at high risk of developing specific genetic conditions, to testing of healthy people with the rationale of facilitating life planning. This idea has been taken to the extreme with initiatives such as the BabySeq project, exploring the medical, behavioural and economic impacts of integrating genome sequencing into the care of healthy newborns [61].

Case Study 4 **Grime on the crystal ball (fictional case based on Moscarello et al. [60])**

A healthy medical student was given a direct-to-consumer genetic test for Christmas, and explored the raw data from this test using an online interpretation programme, finding a variant in *MYBPC3* that was predicted to cause hypertrophic cardiomyopathy. He was understandably worried by this result, taking time off university as he came to terms with it, and giving up running, which he used to really enjoy.

He was seen in a hypertrophic cardiomyopathy clinic and had an expert cardiology assessment including ECG, echocardiogram and review of his family history. He was found to have no clinical evidence of hypertrophic cardiomyopathy, and further genetic testing showed that he did not actually have the disease-causing *MYBPC3* variant that the online interpretation programme had identified. However, he continued to feel anxious about his risk of heart problems and decided to give up running permanently.

**Key messages**

- Information provided from direct-to-consumer testing may be unreliable, especially where online interpretation programmes are used to further explore the raw data from the test: the level of quality control may be very different from that of accredited genetic laboratories, increasing the likelihood of false positives, false negatives and sample mix-up.
- Many direct-to-consumer genetic tests involve no meaningful pre-test counselling – people are often totally unprepared for the information that might come out of such testing (and are unaware that it might be wrong).

## Genetic information as family information

The familial nature of genetic information has always generated discussion as to how to respect the confidentiality of individual patients while ensuring that their close relatives have access to information that may be relevant for their own health and life choices. Clinical guidance in this area has increasingly taken the stance that genetic information should be confidential to families, not individuals (though the personal consequences of having a genetic change for a given individual should be confidential to them alone) [62].

The consequences of this shift are still being navigated in the clinical setting – research indicates that patients often see genetic information as belonging to their family rather than exclusively to them [63], but healthcare professionals are often reticent about taking a familial approach to the confidentiality of genetic information in practice, worrying that this stance could disrupt family dynamics or erode patient trust in the health service [64]. A recent BMJ poll which asked, ‘Are there situations when sharing a patient’s genetic information with relatives without consent is acceptable?’ demonstrated the current split in opinion, with 51% of respondents answering ‘yes’ and 49% ‘no’ [65]. The personal versus familial nature of genetic information is currently being tested in the courts via the ABC case, which centres around non-disclosure of genetic risk to the daughter of a patient with Huntington’s disease [66].

## Treatment for genetic disorders

One of the most exciting recent developments in genetics and genomics is the prospect of treatment for an increasing number of genetic conditions. However this topic has to be treated with caution as the practical reality for many patients and families is that though promising research is ongoing, meaningful treatment is not possible in many cases. Even in situations where evidence-based treatments have been developed, the expense of many of these therapies risks making them inaccessible.

Many different approaches have been taken to try to treat genetic conditions. Gene therapy, which involves delivering functional genetic code, is one approach but its success has been widely variable, often due to difficulty in developing vectors that can deliver genetic material into affected tissues at sufficiently high levels without being destroyed by the immune system. In certain situations this approach can be highly effective, for example promising results have been achieved in various eye conditions, likely because eyes are small and easily accessible, and have a privileged relationship with the immune system [67]. In cases aiming to deliver gene therapy to a wider area, such as the lungs or the muscles, treatment attempts have generally proved more challenging [68,69].

Other approaches include use of small molecules to modify various steps in the pathway from gene to functional product. For example, Eteplirsen aims to treat Duchenne muscular dystrophy in certain patients by influencing splicing machinery to skip exon 51 from mature *DMD* mRNA, restoring a more functional reading frame so that a shortened version of dystrophin can be successfully translated [70]. Ivacaftor potentiates the action of CFTR channels in some patients with cystic fibrosis (G551D pathogenic variant) [71]. Enzyme replacement therapy is being trialled to treat children with mucopolysaccharidoses, for example idursulphase infusions in mucopolysaccharidosis type 2 [72].

While lots of these therapies are very exciting and show demonstrable changes at the molecular level in clinical trials, these cellular changes do not always clearly translate into improvements in clinically relevant outcomes. The therapies are also often hugely expensive, which raises very difficult ethical questions regarding whether limited resources should be spent on such treatments where there is often only limited proof of clinical efficacy.

However the increasing possibility of future treatments for genetic conditions is influencing clinical decisions around the care of very ill children. For example, recently nusinersen has shown promise as a treatment for some children with spinal muscular atrophy, but this may begin to raise new questions about whether interventions such as intubation and tracheostomy should be offered to infants with severe spinal muscular atrophy, where previously these would have been considered medically inappropriate [73]. This has consequences for the clinical conversations happening when these diagnoses are made. In the past, breaking news of such a diagnosis might flow naturally into discussions around palliation. The possibility of treatment now creates new options to consider, but also new challenges in considering with parents how best to care for their child [74]. The clinical impact and accessibility of emerging treatments is often very uncertain, but parents may prefer to explore even extremely long-shot treatments over accepting a palliative care pathway route, and may expect or seek crowd funding for experimental treatments for which there is as yet very little, if any, evidence of benefit.

Improving genetic technology has also had a significant impact on fertility services, ranging from pre-implantation genetic diagnosis to mitochondrial donation, offering new options for families affected by genetic conditions [75,76]. Increasing technological capability is set to extend the theoretically possible range of options – for example last year a group in China used the CRISPR/Cas9 system to correct pathogenic variants in the *HBB* and *G6PD* genes in human zygotes [77], though the efficiency and accuracy of the correction procedure was variable. This emerging possibility raises significant ethical issues which need debate. A recent report of the Nuffield Council on Bioethics on genome editing in the context of human reproduction suggested that there may be certain contexts in which this may be ethically acceptable, provided that such interventions were intended to secure the welfare of a person who may be born as a result, and that any such interventions would uphold principles of social justice and solidarity [78].

## Conclusions

Insights from genomic technology have great potential to improve health, but we are currently going through a teething process in learning how to respond to the nebulous information that genomic tests can provide in the clinical setting. In part, this learning process is being driven by patients and families, with patient support groups coming to the fore in an era where we can now make extremely rare diagnoses that link different families across the world, but often have very little information on what this might mean for the future. Our current response to the outcomes from genomic tests is often reactive and ad hoc, partly because we are still learning how to interpret genomic variation and are often unable to gain a consensus on whether genetic variants are clinically significant or not. This situation is exacerbated by the different routes in which genomic information is now accessible – rapid tests to establish diagnosis or plan treatment for patients are now a reality in the real-life clinical setting, but healthy people also have increasing access to commercial tests that claim to provide genetic information to improve health and life planning. This raises particular challenges in the context of a public discourse about genomics that tends to present it as far more predictive and certain than it actually is. Some of the most exciting recent developments in genomic medicine relate to potential future treatments and reproductive options for people and families affected by rare genetic conditions. However hurdles relating to treatment efficacy and optimal timing of treatment, mean that we need to keep these advances in perspective and consider how to research potential treatments responsibly, avoiding creating hype that undermines the ability of families to make a balanced decision whether or not to participate in this research. It is also important to consider financial sustainability, avoiding situations where useful new treatments are developed that remain inaccessible to the patients who need them on account of their cost. To summarise, the introduction of genomic testing is having a big impact on patient care, but raises various issues that need further study and debate in order to help us maximise the potential benefits of genomic medicine while minimising the possible harms.

## Footnotes

1 Complex chromosome rearrangements, thought to occur due to single catastrophic events where chromosomes ‘shatter’ and are repaired by error-prone mechanisms.

2 Clusters of localised mutations.

## Abbreviations

ACMG: American College of Medical Genetics and Genomics
GA4GH: Global Alliance for Genomics and Health
GWAS: Genome-wide association study
SNP: Single nucleotide polymorphism
MRSA: Methicillin-resistant *Staphylococcus aureus*
